# Identification and differential regulation of microRNAs in response to methyl jasmonate treatment in *Lycoris aurea* by deep sequencing

**DOI:** 10.1186/s12864-016-2645-y

**Published:** 2016-10-10

**Authors:** Sheng Xu, Yumei Jiang, Ning Wang, Bing Xia, Yilong Jiang, Xiaodan Li, Zhengzhi Zhang, Yikui Li, Ren Wang

**Affiliations:** 1 Institute of Botany, Jiangsu Province and Chinese Academy of Sciences, Nanjing, 210014 China; 2 National Center for Soybean Improvement/Key Laboratory of Biology and Genetic Improvement of Soybean (General, Ministry of Agriculture), Nanjing Agricultural University, Nanjing, 210095 China; 3 National Center for Soybean Biotechnology and Division of Plant Sciences, University of Missouri, Columbia, MO 65211 USA

**Keywords:** Amaryllidaceae alkaloids, Deep sequencing, Degradome, *Lycoris aurea*, Methyl jasmonate, MicroRNA

## Abstract

**Background:**

*Lycoris aurea* is a medicine-valuable and ornamental herb widely distributed in China. Former studied have showed that methyl jasmonate (MJ) treatment could increase the content of glanthamine-a worldwide medicine for symptomatic treatment of Alzheimer’s disease in genus *Lycoris* plants. To explore the possible role of miRNAs in the regulation of jasmonic acid signaling pathway and uncover their potential correlations, we investigated the expression profiles of small RNAs (sRNAs) and their targets in *Lycoris aurea*, with MJ treatment by using next-generation deep sequencing.

**Results:**

A total of 365 miRNAs were identified, comprising 342 known miRNAs (representing 60 miRNA families) and 23 novel miRNAs. Among them, 143 known and 11 novel miRNAs were expressed differently under MJ treatment. Quantitative real-time PCR of eight selected miRNAs validated the expression pattern of these loci in response to MJ treatment. In addition, degradome sequencing analysis showed that 32 target genes were validated to be targeted by the 49 miRNAs, respectively. Gene function and pathway analyses showed that these targets such as auxin response factors (ARFs), squamosa promoter-binding like (SPL) proteins, basic helix-loop-helix (bHLH) proteins, and ubiquitin-conjugating enzyme E2 are involved in different plant processes, indicating miRNAs mediated regulation might play important roles in *L. aurea* response to MJ treatment. Furthermore, several *L. aurea* miRNAs associated with their target genes that might be involved in Amaryllidaceae alkloids biosynthehsis were also analyzed.

**Conclusions:**

A number of miRNAs with diverse expression patterns, and complex relationships between expression of miRNAs and targets were identified. This study represents the first transcriptome-based analysis of miRNAs in *Lycoris* and will contribute to understanding the potential roles of miRNAs involved in regulation of MJ response.

**Electronic supplementary material:**

The online version of this article (doi:10.1186/s12864-016-2645-y) contains supplementary material, which is available to authorized users.

## Background

*Lycoris aurea* (L’ Hér) Herb is a popular ornamental species of Amaryllidaceae plant widely distributed in China. It belongs to the genus *Lycoris* and the bulbs are very durable, tolerating the extremes of drought and waterlogging, as well as poor soil conditions. *Lycoris* species possess the plentiful flower colours and shapes [1], and are also important of medical values [2, 3]. A variety of secondary metabolites known as Amaryllidaceae alkaloids are found in *Lycoris*, exhibiting many important pharmacological properties, such as immunostimulatory, anti-tumoral, anti-viral and anti-malarial activities [4, 5]. For example, galanthamine, as a selective and reversible acetylcholinesterase inhibitor [6, 7], has been used worldwide in medicine for symptomatic treatment of Alzheimer’s disease [8]; lycorine, a pyrrolophenanthridine alkaloid, has been demonstrated to display very promising anti-tumor activity in animal and human cell lines [9, 10].

Many detailed insights in biosynthetic steps of Amaryllidaceae alkaloids production are revealed by the biochemical approaches labeling intermediates. In general, Amaryllidaceae alkaloids are regarded as derivatives of the common precursor 4′-*O*-methylnorbelladine via intramolecular oxidative phenol-coupling [11, 12]. There are three different groups of Amaryllidaceae alkaloids that are biosynthesized by three modes of intramolecular oxidative C–C phenol coupling (*para-ortho*’, *para-para*’ and *ortho-para*’). The *para-ortho*’ oxidative coupling leads to galanthamine, *para-para*’ coupling gives rise to marithidine or crinine derivatives, and *ortho-para*’ coupling yields lycorine [12]. Additionally, in some plants of Amaryllidaceae family, the improved production of Amaryllidaceae alkaloids was observed when treated with methyl jasmonate (MJ), or other elicitor like ethylene [13–17].

Jasmonic acid (JA) system is a key component in the complex plant hormone signaling systems. It is biosynthesized from α-linolenic acid by the octadecanoid pathway and oxygenation of α-linolenic acid is the initial step. The oxygen has to be inserted in the C-13 position by a lipoxygenase (LOX) to form (13S)-hydroperoxy-octadecatrienoic acid (13-HPOT) [18]. 13-HPOT is then utilized as substrates in the following reaction initiated by allene oxide synthase (AOS) and further converted to (9S, 13S)-12-oxo-phytodienoic acid [(9S, 13S)-OPDA] by allene oxide cyclase (AOC) [18]. For further conversion, OPDA is translocated from chloroplasts to peroxisomes, where JA biosynthesis continues and a series of enzymes including OPDA reductase 3 (OPR3), acyl-CoA oxidase (ACX), multifunctional protein, and L-3-ketoacyl CoA thiolase are involved in. JA and its derivatives, collectively referred to as jasmonates, are important signal molecules detected in a wide spectrum of plant species and function on a lot of biological processes including growth inhibition, senescence, wound response, plant defense and secondary mechanism [18, 19]. For example, it was first demonstrated that an endogenous rise in JA levels upon elicitation with a rough yeast elicitor was associated with the induction of alkaloid synthesis in plant cell cultures [20]. Later, by inducing taxoid biosynthesis with MJ in *Taxus cuspidata* cells, two cytochrome P450 cDNA clones that encoded hydroxylases of the taxol biosynthetic pathway were identified [21]. Additionally, exogenous MJ elicits massive accumulation of caffeoylputrescine in tomato and this introduction is tightly controlled by the constitutive activation of the jasmonate signaling pathway [22]. Broadly, three major classes of plant secondary metabolites defined as the terpenoids, alkaloids and phenylpropanoids were induced by jasmonates [18, 23].

In recent years, plant sRNAs are getting more and more attention for their regulatory roles in growth, development as well as defense process [24, 25]. They are classified into two major categories: small interfering RNAs (siRNAs) and microRNAs (miRNAs) [26]. miRNAs negatively modulate the expression of a wide range of genes, at the post-transciptional levels by directing the mRNA cleavage or by repressing translation [27]. miRNAs have been shown to play crucial roles in plant responses to a variety of abiotic and biotic stresses, such as nutritional deficiency [28], drought [29], salinity [30], cold [31], heat [32], heavy metal stress [33], and disease [34]. Additionally, many lines of evidences support that miRNA-mediated gene regulation also plays a significant role in plant secondary metabolites biosynthesis [35, 36]. Previously, by using high-throughput sequencing, the taxoid elicitor MJ-regulated *Taxus chinensis* and *Arabidopsis* miRNA expression was observed [37, 38]. However, little is sensed about MJ-mediated mechanism on miRNAs.

For investigating roles of miRNAs in the regulation of biological process in *L. aurea*, we employed high-throughput sequencing technology to survey small RNA pools in *L. aurea* experimentally treated with MJ. Our results indicate a complex and diverse small RNA population exists in *L. aurea*, thereby providing a meaningful database to microRNA study in *Lycoris* species. Moreover, our observation suggests that there be a correlation between the MJ-elicited miRNAs and *L. aurea* biological process such as Amaryllidaceae alkaloids biosynthesis via a possible transcriptional or post-transcriptional regulation.

## Results and discussion

### Transcriptome sequencing and *de novo* assembly analysis

We generated transcriptome database with the Illumina Hiseq2000 system, after sequencing the two *L. aurea* cDNA libraries from the MJ-treated sample (MJ 100) and its untreated control (CK). In total, each library comprises about 53.45 million raw sequence reads. After removing poly (A) tails, short and low-quality tags, and adaptor contamination, 26,809,842 (CK) and 25,874,478 (MJ100) clean reads with a total of 2,412,885,780 and 2,328,703,020 nucleotides were obtained respectively, for the two pools. Due to the unavailability of the full genome sequences of *L. aurea*, the assembly software Trinity [39] was performed for *de novo* assembly of all the 52,684,320 clean reads. Combined with our GS FLX titanium platform of 454 pyrosequencing transcriptome data reported previously [40], the entire integrated transcriptome was subsequently used to analyze *L. aurea* sRNAs and degradome libraries.

### High-throughput sequencing of CK and MJ-treated small RNA libraries

**Fig. 1:**
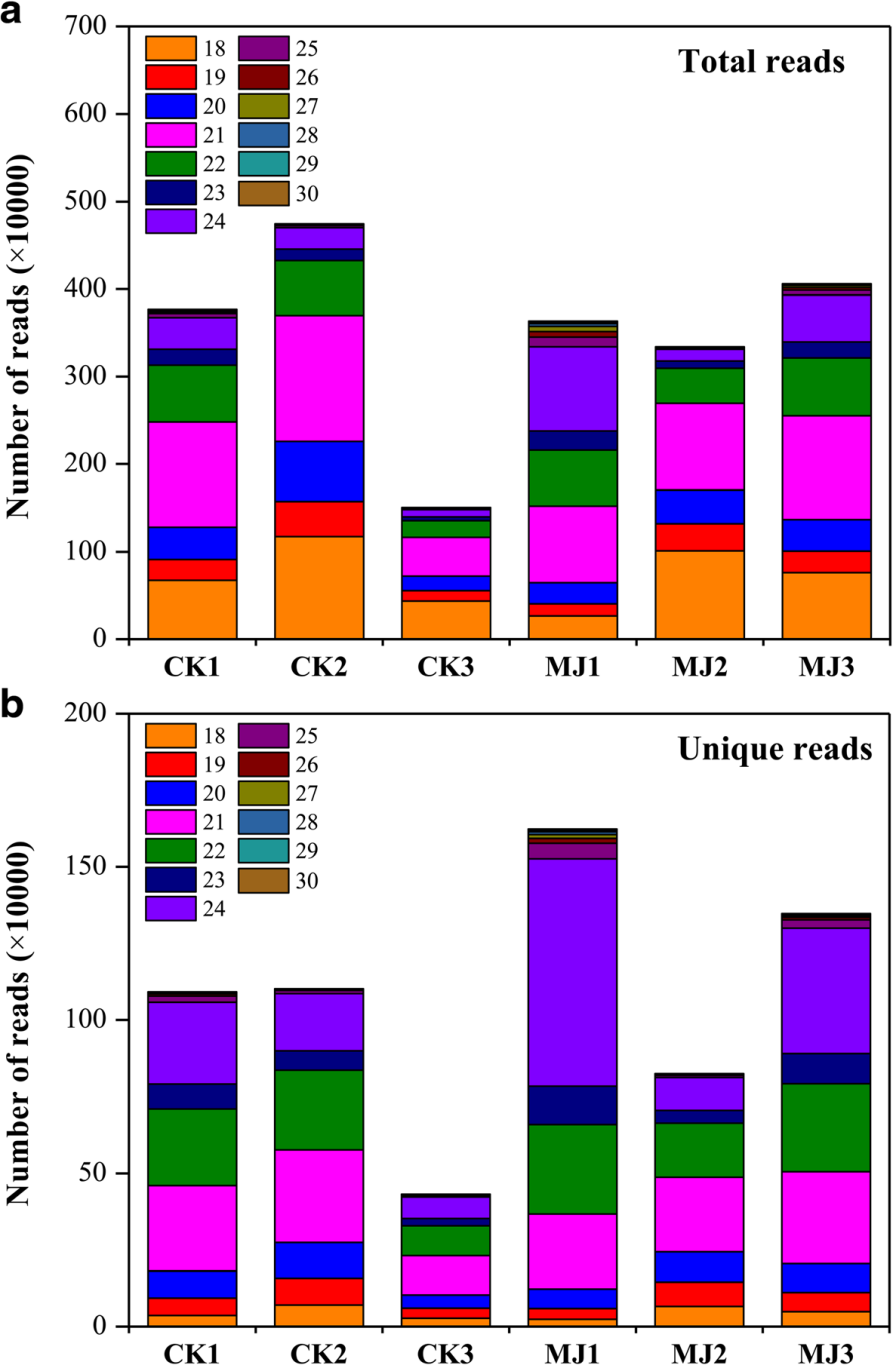
The length distribution of total (**a**) and unique (**b**) small RNA reads from MJ-free (CK) and MJ-treated libraries of *L. aurea*. Number of high-quality filtered total reads (after quality control and removal of redundant reads) of size 18–30 nt in different samples is shown

To perform a wide discovery of miRNAs in *L. aurea*, we sequenced six small RNA libraries constructed from control (CK1, CK2 and CK3) and MJ-treated (MJ1, MJ2 and MJ3) samples using the Illumina sequencing platform. A total of more than 29.8 million sequence reads were generated from all the samples, in the range 3.0–6.9 million for individual sample (Additional file 1: Table S1). After filtering out the adapter sequences as well as sequences with low quality, and further removing poly-A sequences and short RNA reads smaller than 18 nucleotides and larger than 40 nucleotides, the total number of clean sequences were reduced to about 21.0 million (Table 1 and Additional file 1: Table S1). The number of unique sRNAs ranged from 0.4 to 1.6 million for individual sample (Table 1). Figure 1 shows size distribution of the sRNAs from the MJ and CK libraries. The size distribution of the filtered sequence reads indicated the high-quality of the data and the majority of the small RNA reads were distributed between 18 to 30 nt. The largest fraction of total sRNAs was 21 nt long in all the samples analyzed (Fig. 1a). This result was consistent with previous reports for other plant species, such as *Taxus mairei* [41], *Panax ginseng* [42], *Pinus contorta* [43], *Vitis vinifera* [44] and *Saccharina japonica* [45]. Additionally, size distribution analysis of the 1,781,274 unique small RNA sequences of all the samples showed that the 24 nt group was the biggest, which accounted for 27.64 % of total unique sequences (Fig. 1b). It suggests that 24 nt sRNAs are the most diverse in *L. aurea*, which is similar to the results observed in other plant species [42, 46, 47]. Overall, more than 75 % of the unique sRNAs in length were within the range of 21–24 nt. These observations were consistent with DICER-LIKE (DCL) protein cleavage products and those reported in previous studies [48–50].

**Table 1:** Statistical analysis of sequencing reads from the CK and MJ100 sRNA libraries in *L. aurea*

Category	CK1		CK2		CK3		MJ1		MJ2		MJ3	
	Total	Unique	Total	Unique	Total	Unique	Total	Unique	Total	Unique	Total	Unique
Clean reads	3,776,867	1,094,182	4,648,051	1,103,602	1,513,368	434,141	3,680,211	1,634,253	3,342,240	825,583	4,076,785	1,353,202
miRNA	149,287	402	200,761	408	59,857	322	118,396	318	134,827	389	158,220	385
rRNA	133,671	25,564	159,974	24,172	51,309	13,645	155,927	32,284	118,064	18,606	156,015	26,776
tRNA	69,481	10,396	101,905	10,616	31,025	5,776	102,000	15,393	57,567	7,849	84,561	12,534
snRNA	13,089	5,210	11,528	4,169	4,171	2,050	11,902	6,156	8,452	3,514	12,324	5,278
Cis-reg	6,266	2,201	8,348	2,110	2,740	961	7,908	3,629	6,507	1,594	7,601	2,757
repeats	113	99	86	73	37	37	320	286	69	58	174	151
gene	1,584,018 (42.94 %)	166,386 (15.21 %)	2,188,034 (47.07 %)	192,626 (17.45 %)	735,521 (48.60 %)	82,958 (19.11 %)	1,106,681 (30.07 %)	206,407 (12.63 %)	1,611,524 (48.22 %)	141,584 (17.15 %)	1,623,832 (39.83 %)	196,857 (14.55 %)
others	26,637	6,610	34,754	6,723	10,591	2,976	27,716	9,395	22,419	5,020	28,787	7,799
unannotated	1,794,305	877314	1,942,661	862,705	618,117	325,416	2,149,361	1,360,385	1,382,811	646,969	2,005,271	1,100,665

As a complete *L. aurea* genome sequence is not yet available, we mapped the sequenced sRNAs to the *L. aurea* transcriptome. A total of 8,849,610 reads (with an average of 42.07 % total small RNA) representing 986,818 unique reads from all the samples were mapped to the transcriptome of *L. aurea* (Table 1 and Additional file 1: Table S1). However, only a small proportion of the unique sRNAs (15.31 %; Table 1) mapped to the transcriptome of *L. aurea*. Former study has found that a majority of miRNA genes in plants were located in intergenic regions [51]. For example, among 26 miRNA sequences identified in *Ectocarpus*, 17 miRNA sequences are located in introns, eight located in 335 intergenic regions, and one in a local antisense to a transposable element [52]. So it is possible that a high proportion of miRNA loci in *L. aurea* is also located in the intergenic region and/or introns rather than in the gene exons in *L. aurea*.

### Identification of conserved and novel miRNAs in *L. aurea*

**Fig. 2:**
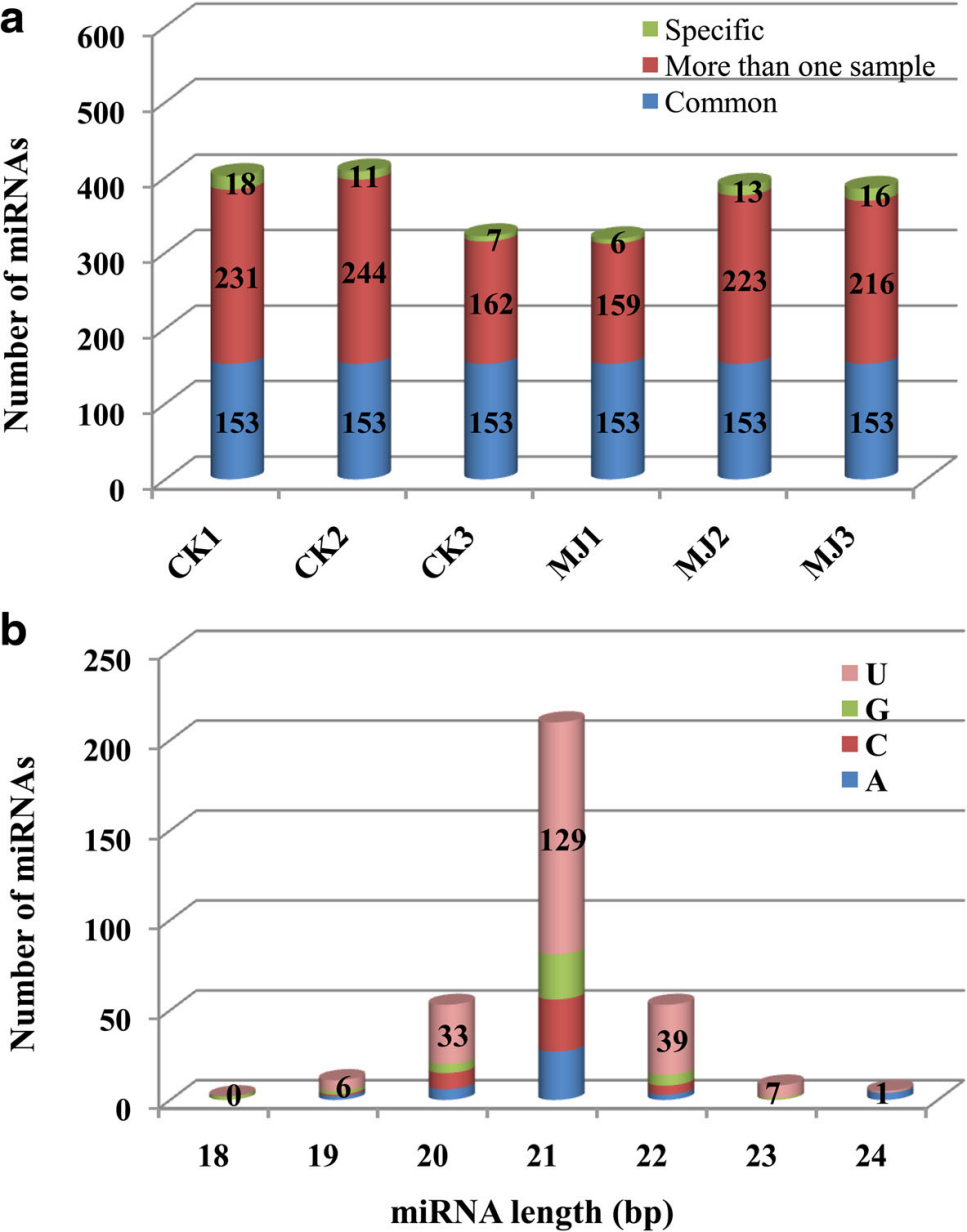
microRNA prediction in the samples of *L.aurea*. **a** Number of miRNAs identified in six samples are shown along with their specificity. The number of miRNAs identified in all the samples (common), more than one sample, and those specific to each sample are given. **b** The length distribution of miRNAs and the identity of the first nucleotide at 5′ residue

According to alignment of the remaining sequences from the six libraries against all conserved/known miRNAs in miRBase (Release 20.0) with no mismatches and without gaps, a total of 821,348 reads from all the samples were mapped on the miRBase plant miRNAs, which resulted in the identification of a total of 402, 408, 322, 318, 389, and 385 unique conserved miRNAs from CK1, CK2, CK3, MJ1, MJ2, and MJ3, respectively (Table 1). Many of these miRNAs were present in one or more samples analyzed (Fig. 2a). A comparative analysis of miRNAs identified from each sample led to the identification of a total of 342 non-redundant distinct conserved miRNAs of size 18–24 nt in *L. aurea*. Of 342 miRNAs, 153 were present in all the samples analyzed (Fig. 2a). A significant fraction of the miRNAs was also identified only from a specific sample (Fig. 2a). The size distribution analysis showed the predominant (61.40 %) representation of 21-nt-long miRNAs (Fig. 2b). About both 15.50 % of miRNAs were 20-nt- and 22-nt-long, whereas only 7.60 % was of the rest of the miRNAs (Fig. 2b). Further, most miRNAs of different lengths harboured a uridine residue at the 5′-end (Fig. 2b). Higher abundance of 21-nt-long miRNAs with uridine as a 5′ terminal nucleotide in *L. aurea* correlates with other plant species reported before [48]. The 21-nt-long miRNA with 5′-uridine is a characteristic feature of DCL1 cleavage and AGO1 association, which has been found in most known miRNAs [49, 53, 54]. Further, the presence of multiple numbers of DCL and AGO proteins can produce miRNAs with different lengths, first nucleotide specificity and diverse functionality [53, 55–57].

We also analyzed the nucleotide composition of mature miRNAs in *L. aurea*. Most (77.78 %) of the miRNAs had a GC content within the range of 41–60 % (Additional file 2: Figure S1). The average GC content of mature miRNAs in *L. aurea* (51.89 %) was similar to that observed in *Populus* (50 %), grapevine (50 %) and maize (52 %) [50]. It has been suggested that the GC content of miRNAs can be used as critical parameter for determining their biological roles [58].

Additionally, a total of 7.0 % reads matched to structural non-coding RNAs (snRNA, tRNA, and rRNA), repeat sequences and other sequences (Table 1). After removal of these reads, the 9,892,526 unannotated reads (about 47.20 % of total sRNAs) representing 5,173,454 unique reads were used for novel miRNA prediction using the miRDeep2 pipeline. The processing involved extraction of precursor sequences from the transcriptome by extending mapped reads in the flanking regions, and its potential to form a stem-loop secondary structure. After processing, a total of only 23 novel miRNA precursor candidates for CK and MJ libraries were obtained (Additional file 3: Table S2). We also found that the average expression level of the novel miRNAs was much lower than that of the conserved miRNAs (196 reads per miRNA versus 2402 reads per miRNA), implying that most of novel miRNAs are young miRNAs with imprecise processing and lack of targets [57]. Our sequence analysis showed that the putative pre-miRNAs of each library greatly varied from 55 to 372 nucleotides in length (Additional file 3: Table S2). Some of the stem-loop secondary structures of predictive pre-miRNAs of *L. aurea* could be found in Additional file 2: Figure S2. In addition, miRDeep2 implemented other criteria, such as seed conservation and presence of miRNA* evidence, which ensured high-confidence prediction of miRNAs.

### Identification of miRNA families and expression analysis of candidate miRNAs in *L. aurea*

**Fig. 3:**
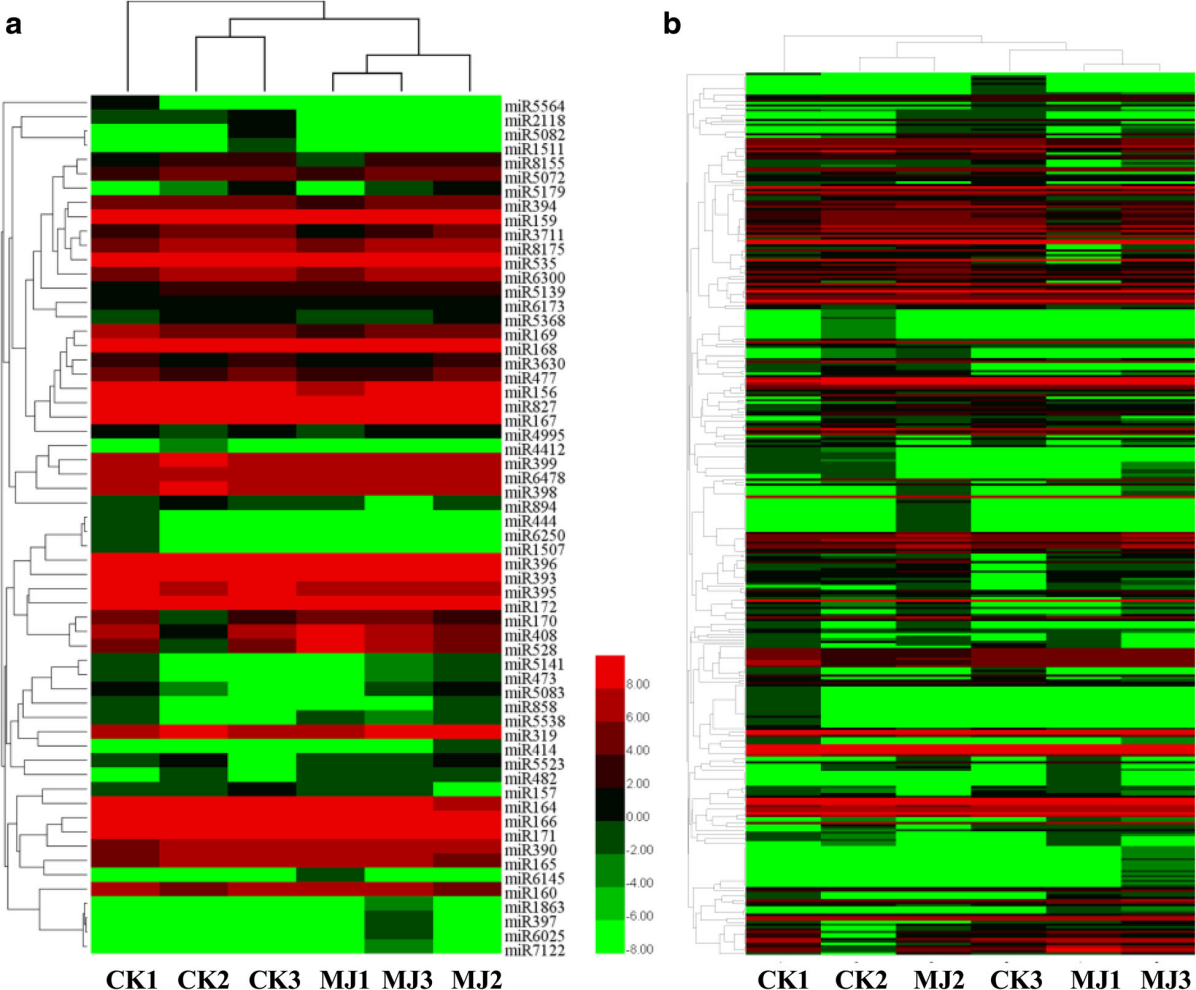
Expression analysis of *L.aurea* miRNA. Heatmap showing miRNA family (**a**) and conserved miRNA (**b**) expression patterns

Based on sequence similarity, we clustered all the identified *L. aurea* miRNAs into families. 342 conserved miRNAs were clustered into 60 known families and the number of miRNAs in each family varied (Additional file 4: Table S3). Among them, a few (32) of miRNAs were represented only by a single member (Additional file 4: Table S3). This result indicates a kind of diversity of miRNAs appeared in *L. aurea*. Moreover, 28 miRNA families out of 310 evolutionarily conserved miRNAs were represented by more than one member, and most of the families (20) comprised at least five members. Such large gene families have also previously been reported in plants [50, 51, 59, 60]. Heatmap by HemI software showing conserved miRNA and corresponding miRNA family expression patterns were also presented. As shown in Fig. 3a, for miRNA family expression, three samples for CK and three samples for MJ were clustered into one group respectively, while the clustering of each miRNA expression showed a different clustering pattern (CK1, CK2 and MJ2 clustering in one group; CK3, MJ1 and MJ3 clustering in one group) (Fig. 3b). Furthermore, we performed principal components analysis (PCA) to assess the variability of samples (Additional file 2: Figure S3). The results showed a similar tendency appeared in Fig. 3b.

Among the 60 known miRNA families, 20 differentially expressed miRNA families were identified according to the average expression levels between CK and MJ (Additional file 4: Table S3). Of these, ten miRNA families were up-regulated in MJ100 compared with CK, whereas ten miRNA families showed down-regulated patterns (Additional file 4: Table S3). MJ has been proven to play a key role in changing small RNA expression profile in plants [37, 38, 50]. For example, 14 miRNAs from seven different families (miR156, miR168, miR169, miR172, miR396, miR480 and miR1310) were down-regulated whereas three miRNAs from two families (miR164 and miR390) were up regulated in Chinese yew [37]. Interestingly, among above mentioned miRNAs, we observed no miRNA was induced by MJ (Additional file 4: Table S3), which exhibit the same expression pattern under MJ treatment in *Arabidopsis* [38]. In this study, the average expression of miR398 in all the samples was down-regulated by 1.33-fold in MJ treatment when compared with the CK (Additional file 4: Table S3). It has been shown that miR398 play an important role in the response to the various abiotic stresses. For example, down-regulation of *Arabidopsis* miR398 in response to oxidative stresses is important for two posttranscriptional CSD mRNA accumulation and oxidative stress tolerance [61]. Heat stress rapidly induces *Arabidopsis* miR398, and this induction triggers a regulatory loop that is critical for thermotolerance [32]. Under freezing stress, *Arabidopsis* miR398 was repressed and *Chrysanthemum dichrum* ICE1 [INDUCE OF C-REPEAT BINDING FACTOR (CBF) EXPRESSION 1] regulated freezing tolerance of *Arabidopsis* partly through the miR398-CSD pathway [62]. On the other hand, it has been performed that JA acts with salicylic acid to confer basal thermotolerance in *Arabidopsis thaliana* [63]. Recently, jasmonate functions as a critical upstream signal of the ICE-CBF/DRE BINDING FACTOR1 (DREB1) transcriptional pathway to positively regulate *Arabidopsis* freezing tolerance has also been revealed [64]. Based on these results, we could speculate that miRNA398 probably regulates a complex process in the response to MJ in *L. aurea*. Additionally, we also observed that the average expression of miR528 in all the samples was up-regulated by 2.33-fold in MJ treatment when compared with the CK (Additional file 4: Table S3). Evidence has proven that constitutive expression of miR528, a conserved monocot-specific small RNA alters plant development and enhances tolerance to salinity stress and nitrogen starvation [65]. It also involves in the regulation of arsenite tolerance [66].

On the other hand, a few miRNA families, such as miR166, miR396, and miR159, showed extraordinarily high expression levels in all the libraries, whereas others were present in much lower abundance. For example, miR159 family was the most abundant, with a total of 134,227 (CK) and 127,914 (MJ100) reads accounting respectively (Additional file 4: Table S3). We also noticed that miR156, which has been shown to be involved in the regulation of flowering time and floral development [67, 68] appeared decreased but not significant expression changes in MJ treatment when compared with the CK (Additional file 4: Table S3). miR172, another small RNA regulating flowering time, showed almost no differential expression changes under MJ treatemt in *L. aurea* (Additional file 4: Table S3).

**Fig. 4:**
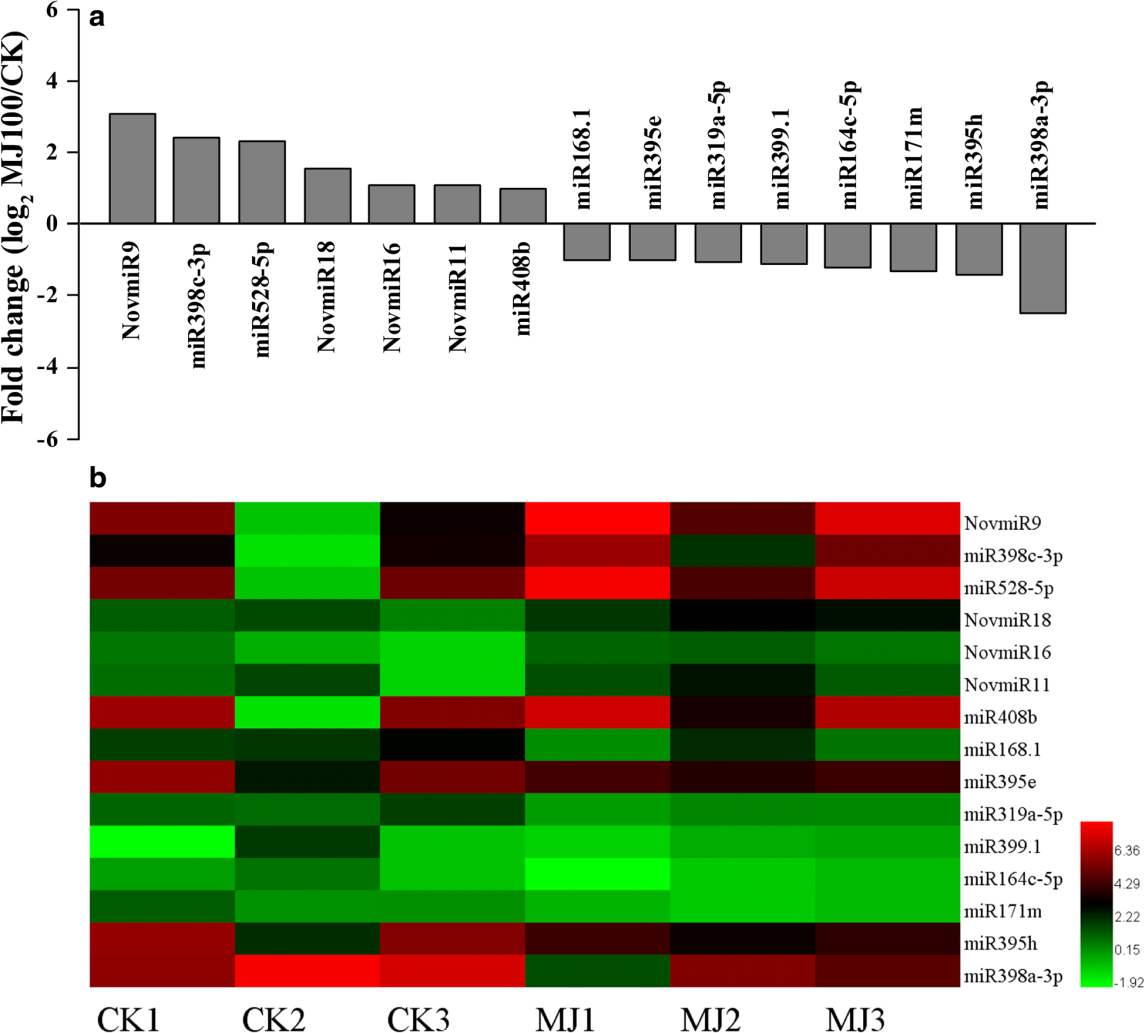
Validation and comparative relative expression of selected conserved and novel miRNAs between the CK and MJ100 libraries in *L. aurea.* **a** The average expression levels of CK and MJ 100 libraries. **b** Heatmap showing expression profile of differently expressed miRNAs in each sample. NOTE: miRNAs of each sample with reads count >1, FDR <0.01, and |Fold change| >1.0 are shown

By calculating log_2_ RPM value, the expression level of the miRNAs including novel miRNAs (Additional file 5: Table S4) was also identified (Fig. 3b). In all, 143 out of 342 conserved miRNAs and 11 out of 23 novel miRNAs were identified as differently expressed miRNAs based on the average expression levels of CK and MJ samples (Additional file 4: Table S3; Additional file 5: Table S4). Among them, most are down-regulated in response to MJ. After restricting our analysis to differentially expressed miRNAs with the following three criteria: Read counts of each sample >1, FDR <0.01 and |Fold change| >1, 15 differentially expressed miRNAs indicating 7 up-regulated and 8 down-regulated miRNAs were observed after the MJ100 treatment (Fig. 4 and Additional file 6: Table S5). These data suggest that the differential regulation of miRNAs account for functions in response to the MJ treatment in *L. aurea*.

### qRT-PCR validation of expression profiles of selected miRNAs

**Fig. 5:**
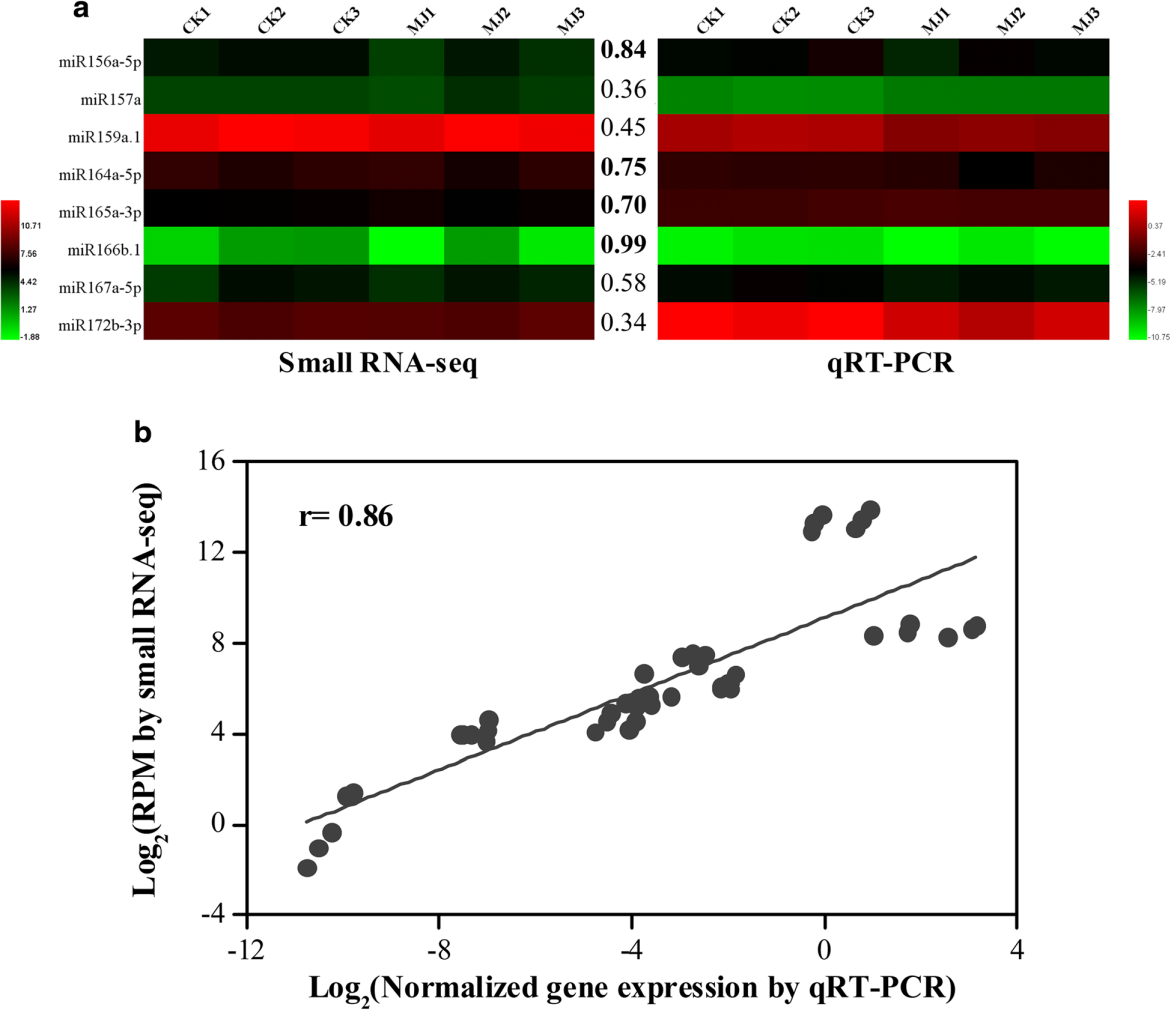
Correlation of gene expression results obtained from small RNA-seq and qRT-PCR analysis for each (**a**) and all (**b**) selected miRNAs in all the samples. The color scales represent log2 transformed normalized expression values for small RNA-seq and qRT-PCR. The values between the two heatmaps represent correlation value between the expression profiles obtained from small RNA-seq and qRT-PCR analysis for each miRNA analyzed. The correlation values above 0.70 are highlighted in bold

To validate the miRNA expression analysis results, we performed qRT-PCR of eight randomly selected conserved miRNAs in all the samples analyzed (Fig. 5). The qRT-PCR data showed a high degree of the agreement with the expression profiles obtained by small RNA sequencing between the CK and MJ100 libraries under MJ treatment at 6 h. For example, a comparative analysis showed similar (correlation >0.70) expression patterns of half (4 of 8) of these miRNAs obtained via small RNA-seq and qRT-PCR analyses (Fig. 5a). Additionally, we also observed a very good concordance in the expression patterns of miRNAs obtained by both small RNA-seq and qRT-PCR as indicated by the overall correlation coefficient (0.86) (Fig. 5b). Similar result was also observed in other plant species [50].

### Identification and annotation of targets for *L. aurea* miRNAs

Because miRNAs function by regulating their target genes [69], identifying the potential target genes of miRNAs is crucially important for understanding miRNA-mediated regulation mechanism during MJ treatment. High-throughput degradome sequencing has been shown to be a valuable and efficient approach to validate and characterize target genes of miRNAs in a variety of plant species [70]. The target genes of the miRNAs under MJ treatment were further studied by degradome library sequencing. In total, ~18.71 million (CK) and ~15.93 million (MJ100) clean reads were obtained. Theses clean reads represent 4 611 090 unique reads from CK and 6 626 101 from MJ100 degradome library (Additional file 7: Table S6). A total of 2,848,018 (61.76 %) and 4,190,778 (63.25 %) unique reads from CK and MJ100 degradome libraries were mapped to the reference database (Additional file 7: Table S6). After identifying degraded targets for each of the miRNAs, the abundance of each sequence was plotted, and the degradation products were grouped into five categories (0–4) according to their relative abundance (Additional file 8: Table S7). In all, 133 targets for the 108 miRNAs were identified, involving 37 miRNA families and 13 novel miRNAs (Additional file 8: Table S7). Among the miRNA families, 11 targeted a single transcript, and the highest number of targets cleaved by a single miRNA was 11 (miR156). Some mRNA targets (such as CL5262.Contig1l), was possibly targeted by two miRNAs (miR156 and miR157).

**Fig. 6:**
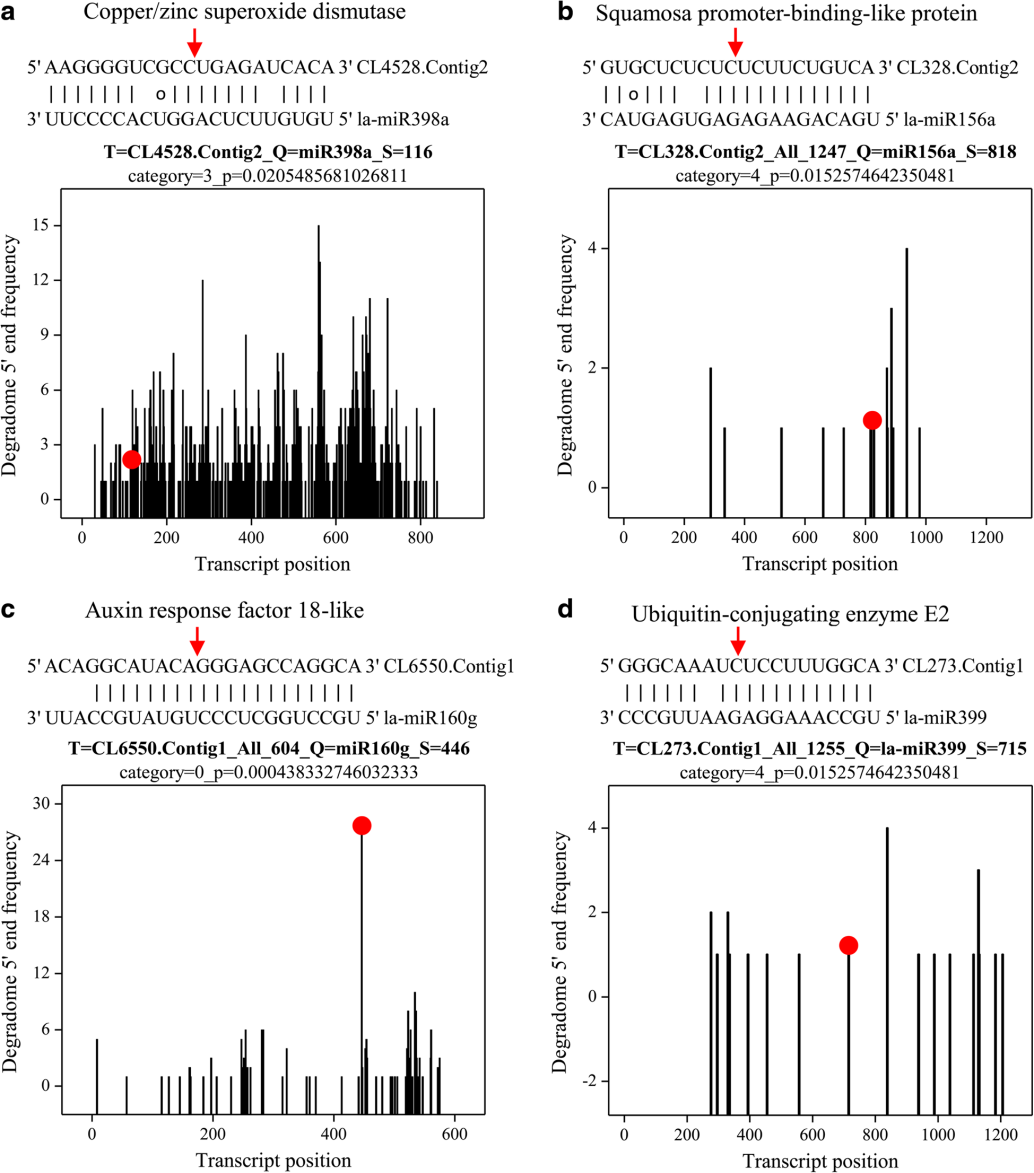
miRNA target alignment and its T-plot validated by degradome sequencing of *L. aurea*. **a** la-miR398a and CL4528.Contig2 (Copper/zinc superoxide dismutase). **b** la-miR156a and CL328.Contig2 (Squamosa promoter-binding-like protein). **c** la-miR160g and CL6550.Contig1 (Auxin response factor 18-like). **d** la-miR399 and CL273.Contig1 (Ubiquitin-conjugating enzyme E2). Both the *arrow* and the *dot* represent the splice site on the miRNA target

A BlastX search of the Nr (non-redundant protein sequences) database showed that the miRNA targets shared homology with other plant proteins (Additional file 8: Table S7). A number of the identified targets for the known *L. aurea* miRNAs were transcription factors, such as the auxin response factor (ARF) family, ethylene-responsive transcription factor, growth-regulating factor, squamosa promoter-binding like (SPL) protein, and GRAS family transcription factor (Fig. 6 and Additional file 8: Table S7). Previous reports have also revealed transcription factors as the predominant targets of miRNAs [48–50, 71, 72]. For example, miR156 and miR157 targeted SPLs, miR172 targeted RAP2 transcirption factors in *L. aurea*, suggesting that these three conserved miRNAs might play significant roles in regulating flowering time and floral development [68]. Some targets also appeared to be involved in signal transduction pathways, such as ARFs and ethylene-responsive transcription factor (Fig. 6 and Additional file 8: Table S7). During *Arabidopsis* adventitious root formation controlled by auxin-regulated JA homeostasis, ARF6 and ARF8, targets of the miR167, are positive regulators, whereas ARF17, a target of miR160, is a negative regulator [73]. Our result also showed that miR167 targeted ARF6 (Additional file 8: Table S7). In addition, JA is also demonstrated to play important roles in plant signal transduction, metabolism, disease resistance, and response to environmental stresses [18]. A few transcripts targeted by conserved miRNAs were involved in plant response to biotic and abiotic stresses, such as those encoding heat shock protein (CL11156.Contig1; miR166 and novmiR19), ubiquitin-conjugating enzyme E2 (CL273.Contig1; miR399) and copper/zinc superoxide dismutase (CL4528.Contig1, CL4528.Contig2 and CL2610.Contig4; miR398, miR528, miR6300, and novmiR9). Ubiquitin–proteasome-mediated proteolysis has also been shown to be involved in jasmonate signaling system [74]. So it could be possible that miRNA-mediated regulation pathway affects jasmonate signaling indirectly.

Previously, the MJ-induced Amarylideceae alkaloids accumulation has been reported [13–15]. According to the transcriptome sequencing, we identified and summarized some genes such as phenylalanine ammonia-lyase, *TYDC*, *O*-methyltransferase (*OMT*), *CYPs*, and *N*-methyltransferase (*NMT*), might be implicated in Amarylideceae alkaloid galanthamine biosynthesis [40, 75]. In the present study, a few of them including cytochrome P450 (CYP) (CL8816.Contig2_All) and tyrosine decarboxylase (TYDC) (CL7028.Contig1) were indentified to be targeted by miRNAs (miR396). TYDC is responsible for the conversion of tyrosine to tyramine [12]. CYPs are able to conduct an intramolecular C–C phenol coupling reaction in plant alkaloid biosynthesis, and at least two CYP-families (CYP80 and CYP719) have acquired this ability [76, 77]. Whether the two *CYPs* identified here are involved in the C–C phenol coupling reaction will need further investigation. Another central feature in the biosynthesis of Amarylideceae alkaloids is methyl transduction at multiple positions of the norbelladine core structure and norgalanthamine, and the reactions are considered to be mediated by OMT and NMT respectively [12, 76]. In our condition, neither miRNA-targeted *OMT* nor *NMT* was found. At the moment, it is difficult to conclude whether miRNAs are also involved in the post-transcriptional regulation of Amarylideceae alkaloids biosynthetic genes, as many *L. aurea* specific miRNA might not be found in the present analysis pipeline.

**Fig. 7:**
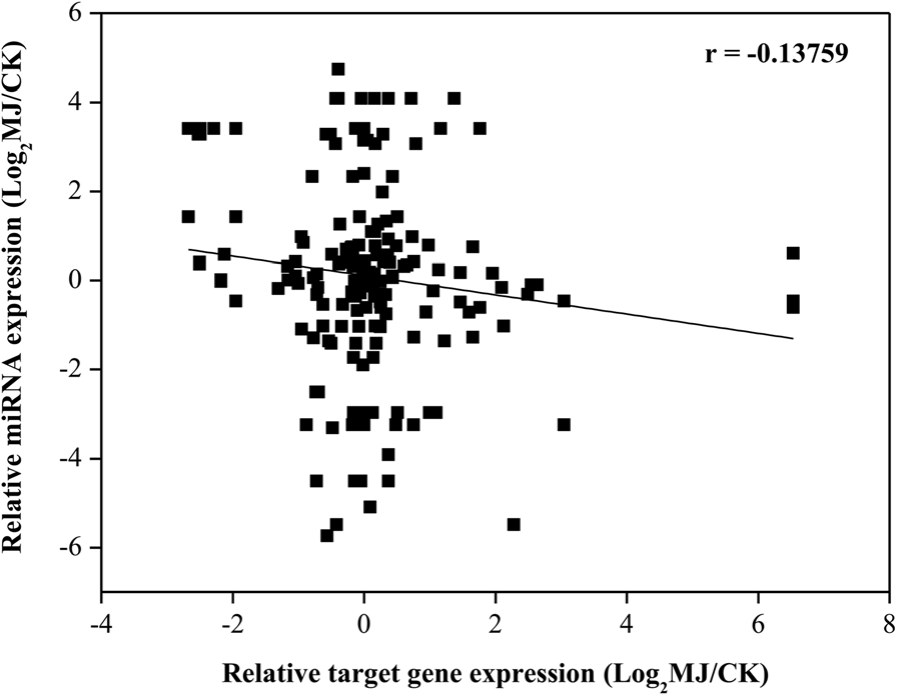
Correlation of expression results obtained from small RNA-seq and RNA-seq analysis for corresponding targets from *L.aurea* CK and MJ samples. A total of 172 data points (average expression levels of miRNAs and targets in the control and MJ-treated samples) are shown in the scatter plot. Each data point represents the log2 normalized expression level obtained from small RNA-seq (y axis) and RNA-seq analyses of targets (x axis)

Plant miRNAs generally direct endonucleolytic cleavage of mRNAs, consistent with the suggestion that plant miRNAs enable rapid clearance of target mRNAs at specific points during plant development [78, 79]. This hypothesis predicts a negative correlation between the expression of a miRNA and its target mRNAs within a given tissue or organ. By comparing the expression levels of the differentially expressed miRNAs with the expression levels of their known and predicted targets, former study has showed that miRNA expression is generally anticorrelated with that of targeted mRNAs [80]. In our study, to investigate the correlation between miRNAs and its target mRNA, a total of 172 data points (average expression levels of miRNAs and their corresponding targets in the control and MJ-treated samples) were shown in the scatter plot (Fig. 7). We also observed a negative but not obvious correlation (correlation = −0.14) in the expression patterns of miRNAs and their targets (Fig. 7).

## Conclusions

A number of miRNAs with diverse expression patterns, and complex relationships between expression of miRNAs and targets were identified. This is the first report on the transcriptome/degradome-wide identification of known and novel miRNAs and their targets in response to MJ in *L. aurea*. The findings could advance our understanding of the functional characterization of miRNAs and their targets in regulating plant response to MJ treatment. This study also provides the first glimpse of the complex regulation of the Amarylideceae alkaloid biosynthetic pathway in *L. aurea*.

## Methods

### Plant culture and MJ treatment

Seeds of *Lycoris aurea* were surface sterilized and germinated on half-strength Murashige and Skoog (MS) medium (pH 5.8) in the dark at room temperature for 10 d. Afterwards, the seedlings were transferred into plastic pots containing a mixture of soil and vermiculite (3:1, *v/v*) and cultured in a growth chamber under 14 h light (25 °C)/10 h dark (22 °C). After 12 months growth, the seedlings were treated with 100 μmol l^−1^ methyl jasmonate (MJ100) for 0, 1, 3, 6, 12 and 24 h, respectively. Seedlings grown in MJ-free solution were used as controls (CK). At each indicated time, three biological replicates for the samples of seedlings were harvested and immediately frozen in liquid nitrogen and stored at −80 °C. The sample exposed to MJ at 6 h was further used for MJ-treated transcriptome, small RNA and degradome sequencing.

### Transcriptome and small RNA sequencing

Total RNA from three biological replicates for the samples was isolated independently using Trizol reagent (Invitrogen) according to the manufacturer’s protocols. Equal amounts of RNA from CK and MJ100 seedlings (6 h) were used for small RNA sequencing. For transcriptome and degradome library construction, the pooled total RNA from three biological replicates of each treatment (CK and MJ) was used. The transcriptome library was prepared using an Illumina TruSeq RNA Sample PrepKit following the manufacturer’s instructions. After removing low-quality reads and reads containing only 3′-RNA adaptors, mRNA transcriptome *de novo* assembly was performed using the SOAP2 program [81].

Small RNA library construction and sequencing were carried out as described previously [82]. Briefly, the small RNA fragments with a length of 18–30 nt were separated and purified by polyacrylamide gel electrophoresis, and ligated first to a 5′ adaptor and then to a 3′ RNA adaptor. The adaptor-ligated sRNAs were subsequently reverse-transcribed to single-stranded cDNA using SuperScript II Reverse Transcriptase (Invitrogen). Finally, deep sequencing was performed on the Illumina Genome Analyzer II (Illumina, San Diego, CA, USA) at Shanghai OE Biotech Company.

### Sequencing data processing and analysis

Small RNA reads were obtained from Illumina HiSeq™ analysis. After removing the low quality reads, reads shorter than 18 nt, reads larger than 40 nt, and trimming adaptor sequences, clean sequencing reads from small RNA libraries were summarized for length distribution and common/specific sequences between samples, and mapped to the *L. aurea* transcriptome sequences. Reads with the same sequence were grouped and termed ‘unique reads’. The clean sequences were first used for mapping to an entire integrated transcriptome of *L. aurea.* The remaining unique sequences were performed for identifying the known/conserved miRNAs from miRBase 20.0 (http://www.mirbase.org/index.shtml) without mismatches and gaps. After that, the sequences matching non-coding RNAs included rRNAs, tRNAs, small nuclear RNAs (snRNAs), and small nucleolar RNAs (snoRNAs) deposited in the Rfam (http://rfam.xfam.org/) and NCBI GenBank (http://www.ncbi.nlm.nih.gov/genbank/) databases were removed. miRDeep2 software [83] was then used to predict novel miRNAs from the left unannotated sRNAs and the stem–loop structures of pre-miRNAs were also constructed.

### Comparison of miRNA expression profiles between CK and MJ-treated *L. aurea* seedlings

The abundance of all miRNAs was normalized to the transcript expression level per million reads (RPM). The log2-transformed RPM value for miRNA expression was used to generate heat map by Heml [84]. For fold change of MJ to CK, average abundance of three MJ samples and three CK samples was calculated respectively. Then the formula: fold change = log_2_ (MJ100/CK) were performed. The significance (*P*-value) of the miRNA expression difference between the CK and MJ100 libraries was calculated with the formula described previously [85]. The false discovery rate (FDR) was also estimated, to determine the threshold of *P*-value. Fold change (|log_2_ (MJ100/CK)| >1) and FDR (<0.01) were combined to identify differentially expressed sequences, which were defined as MJ responsive miRNAs. If the normalized expression was zero, it was adjusted to 0.01.

### Construction and analysis of degradome libraries

Two *L. aurea* degradome libraries were constructed following a previously described method [86]. In brief, poly (A^+^) RNA was isolated from 200 μg of total RNA using the Oligotex mRNA mini kit (Qiagen), and then a 5′ RNA oligonucleotide adaptor containing an *Mme*I recognition site was added to the 5′-phosphate of the poly (A^+^) RNA by T_4_ RNA ligase. After reverse transcription using oligod(T) and PCR enrichment, the PCR products were digested with *Mme*I and ligated to a 3′ double-stranded DNA adaptor. Finally, the ligation products were amplified with 20 PCR cycles, gel purified, and sequenced on an Illumina Genome Analyzer II.

Raw sequencing reads were pre-processed to remove adaptor sequences and low-quality sequencing reads, and only 20–21 nt sequences with high quality scores were retained for subsequent analysis. The degradome reads were mapped to the *L. aurea* transcriptome. Perfect matching sequences were used to validate the predicted miRNA targets using CleveLand4 with default parameters [87]. Alignments with no more than five mismatches and no mismatches at the cleavage site (between the 10th and 11th nucleotides) were retained and scored. Finally, miRNA targets with a *P*-value of ≤ 0.05 were retained.

### qRT-PCR validation

Quantitative real-time PCR (qRT-PCR) for miRNAs was used to validate the MJ responsive miRNAs. Six conserved and six novel miRNAs were selected and subjected to qPCR analysis. Addition of poly (A^+^) tails to sRNAs by poly (A^+^) polymerase and cDNA synthesis were performed using the One Step Primer Script™ miRNA qPCR Starter Kit (TakaRa). PCRs were carried out in a 20 μl reaction mixture consisting of 2 μl of diluted cDNA, 0.2 μM forward and reverse primer, and 10 μl of 2× SYBR Green PCR Master Mix. The reactions were carried out on an iCycler iQ real-time PCR detection system (BIO-RAD) at 95 °C for 30 s, and 40 cycles of 95 °C for 5 s, 56 °C for 15 s, and 72 °C for 20 s. The expression of *U6* snRNA was used as an internal control to normalize for variance in the quantity of RNA and input cDNA. The specificity of each PCR reaction was determined by melting curve analysis. At least two independent biological replicates of each sample and three technical replicates of each biological replicate were analyzed by qRT-PCR. The correlation between sequencing and qRT-PCR based expression analysis results was calculated using the SPSS Version 13.0 software (SPSS Institute, Cary, NC, USA). The primers for selected miRNAs were listed in Additional file 9: Table S8.

## Additional files

Additional file 1: Table S1. Statistics of small RNA sequences from six libraries of *L.aurea. (DOCX 31 kb)*

Additional file 2: Figure S1. Nucleotide composition of miRNAs in *L. aurea*. (A) GC content distribution in mature miRNAs in *L. aurea*. (B) Average GC and AT content in mature miRNAs in *L. aurea*. **Figure S2.** Secondary structure prediction of novel *L. aurea* miRNA precursors (A) NovmiR3 and (B) NovmiR5. **Figure S3.** Principal components analysis (PCA) of miRNA expression of CK and MJ samples in *L. aurea*. (DOCX 1306 kb)
Additional file 3: Table S2. Novel miRNAs predicted by miRDeep2. (XLSX 18 kb)
Additional file 4: Table S3. Conserved miRNA and miRNA families identified by similarity. (XLSX 105 kb)
Additional file 5: Table S4. Expression levels of novel miRNAs in all samples. (XLSX 25 kb)
Additional file 6: Table S5. Validation and comparative relative expression of differentially expressed miRNAs between the CK and MJ100 libraries in *L. aurea. (XLSX 19 kb)*

Additional file 7: Table S6. Abundance (percentage) of different RNAs from *L.aurea* CK and MJ100 degradomes. (DOC 31 kb)
Additional file 8: Table S7. miRNA targets identified from the degradome of CK and MJ100. (XLSX 31 kb)
Additional file 9: Table S8. List of primer sequences used for qRT-PCR experiments. (DOCX 25 kb)

## Abbreviations

(9S, 13S)-OPDA: (9S, 13S)-12-oxo-phytodienoic acid
13-HPOT: (13S)-hydroperoxy-octadecatrienoic acid
ACX: acyl-CoA oxidase
AOC: allene oxide cyclase
AOS: allene oxide synthase
ARF: auxin response factor
bHLH: basic helix-loop-helix
CSD: copper/zinc superoxide dismutase
CYP: cytochrome P450
DCL: DICER-LIKE
DREB1: DRE binding factor1
FDR: false discovery rate
ICE1: induce of C-repeat binding factor (CBF) expression 1
JA: jasmonic acid
LOX: lipoxygenase
miRNA: microRNA
MJ: methyl jasmonate
NMT: *N*-methyltransferase
OMT: *O*-methyltransferase
OPR3: OPDA reductase 3
snRNAs: small nuclear RNAs
sRNAs: small RNAs
siRNAs: small interfering RNAs
snoRNAs: small nucleolar RNAs
SPL: squamosa promoter-binding like
TYDC: tyrosine decarboxylase
