# Supplementary material for: Identification and differential regulation of microRNAs in response to methyl jasmonate treatment in Lycoris aurea by deep sequencing
Source: BMC Genomics. 2016 Oct 10;17:789. doi: 10.1186/s12864-016-2645-y (PMC5057397; doi:10.1186/s12864-016-2645-y)
Supplement: Additional file 1: Table S1. — Statistics of small RNA sequences from six libraries of L.aurea. (DOCX 31 kb) [file 12864_2016_2645_MOESM1_ESM.docx]

**Table S1.** Statistics of small RNA sequences from six libraries of *L.aurea*

| **Library** | Raw (R) | | Clean (T) | | Mapped (M) | |
| --- | --- | --- | --- | --- | --- | --- |
|  | Reads | Unique | Reads  (T/R) | Unique | Reads  (M/T) | Unique |
| **CK1** | 4,284,713 | 1,212,312 | 3,776,867  (88.15%) | 1,094,182 | 1,584,018  (41.94%) | 166,386 |
| **CK2** | 6,118,059 | 1,375,764 | 4,648,051  (75.97%) | 1,103,602 | 2,188,034  (41.07%) | 192,626 |
| **CK3** | 3,041,312 | 859,742 | 1,513,368  (49.76%) | 434,141 | 735,521  (48.60%) | 82,958 |
| **MJ1** | 4,635,321 | 1,883,720 | 3,680,211  (79.40%) | 1,634,253 | 1,106,681  (30.07%) | 206,407 |
| **MJ2** | 4,776,007 | 1,127,492 | 3,342,240  (69.98%) | 825,583 | 1,611,524  (48.22%) | 141,584 |
| **MJ3** | 6,899,990 | 1,938,064 | 4,076,785  (59.08%) | 1,353,202 | 1,623,832  (39.83%) | 196,857 |
| **Total** | 29,755,402 | 8,397,094 | 21,037,522 | 6,444,963 | 8,849,608 | 986,818 |
