# Supplementary material for: Identification and differential regulation of microRNAs in response to methyl jasmonate treatment in Lycoris aurea by deep sequencing
Source: BMC Genomics. 2016 Oct 10;17:789. doi: 10.1186/s12864-016-2645-y (PMC5057397; doi:10.1186/s12864-016-2645-y)

**Figure S1.** Nucleotide composition of miRNAs in *L. aurea*. (A) GC content distribution in mature miRNAs in *L. aurea*. (B) Average GC and AT content in mature miRNAs in *L. aurea*.

**A B**

**Figure S2.** Secondary structure prediction of novel *L. aurea* miRNA precursors (A) NovmiR3 and (B) NovmiR5.


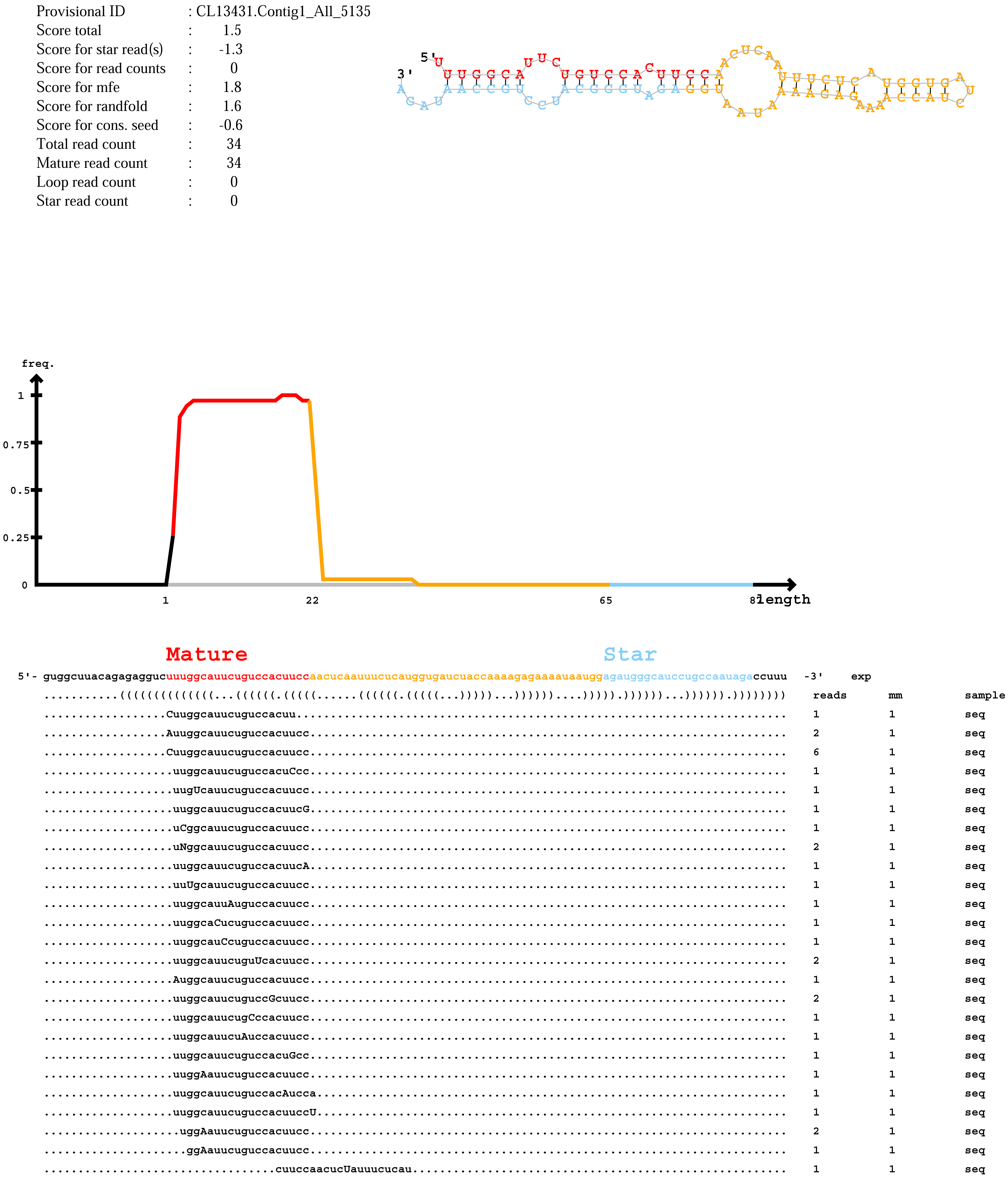


**A**

NovmiR3


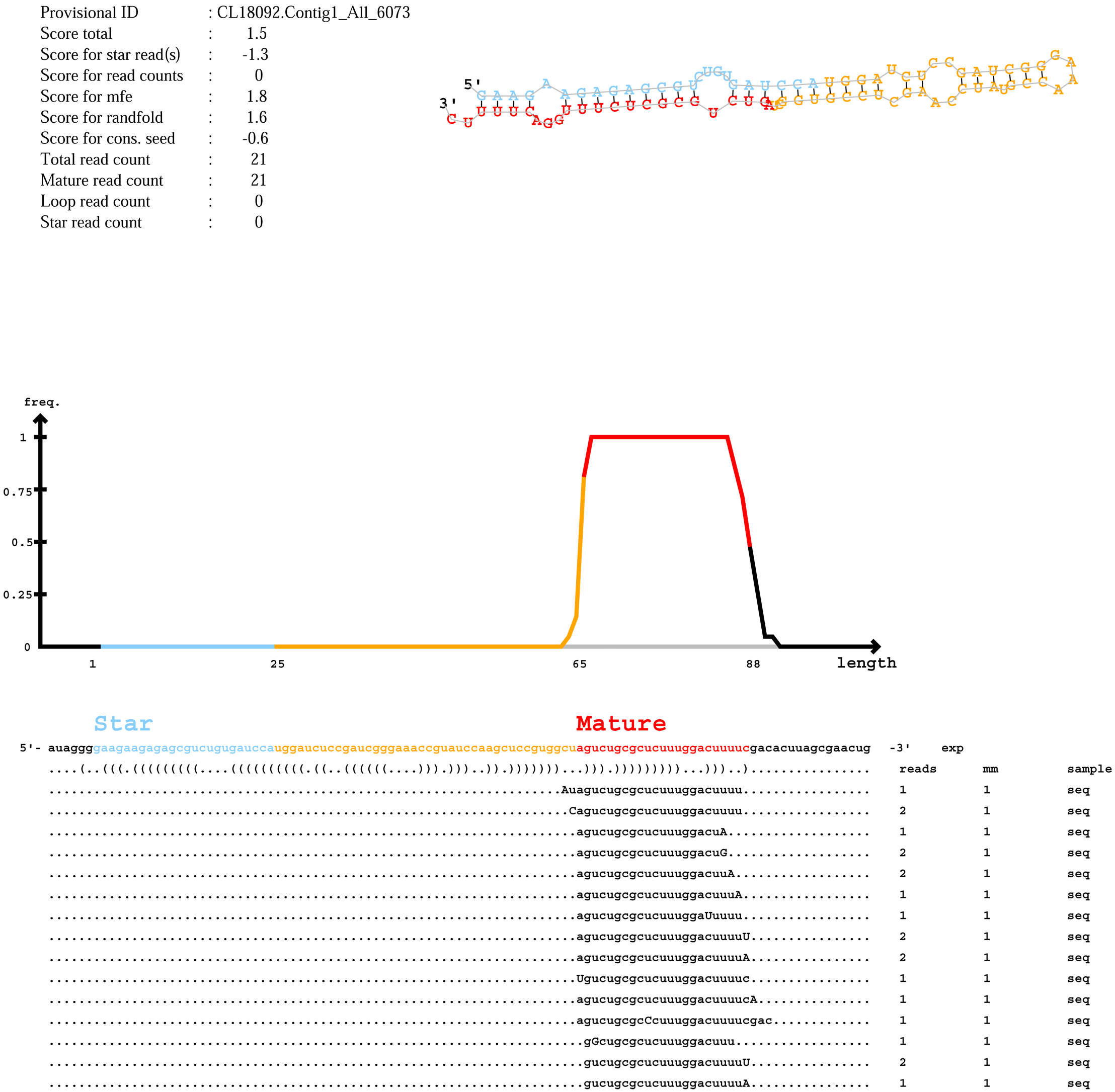


**B**

NovmiR5

**Figure S3.** Principal components analysis (PCA) of miRNA expression of CK and MJ samples in *L. aurea*.


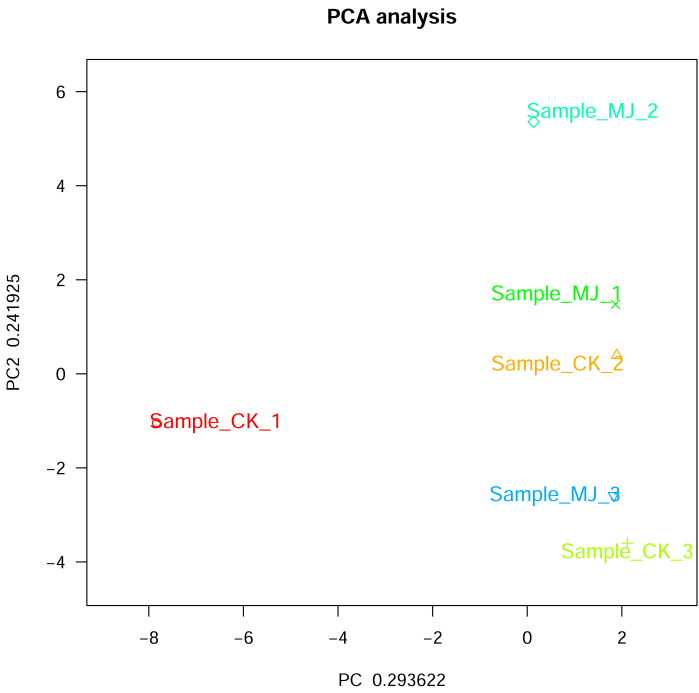

Supplement: Additional file 2: Figure S1. — Nucleotide composition of miRNAs in L. aurea. (A) GC content distribution in mature miRNAs in L. aurea. (B) Average GC and AT content in mature miRNAs in L. aurea. Figure S2. Secondary structure prediction of novel L. aurea miRNA precursors (A) NovmiR3 and (B) NovmiR5. Figure S3. Principal components analysis (PCA) of miRNA expression of CK and MJ samples in L. aurea. (DOCX 1306 kb) [file 12864_2016_2645_MOESM2_ESM.docx]
