# Supplementary material for: Identification and differential regulation of microRNAs in response to methyl jasmonate treatment in Lycoris aurea by deep sequencing
Source: BMC Genomics. 2016 Oct 10;17:789. doi: 10.1186/s12864-016-2645-y (PMC5057397; doi:10.1186/s12864-016-2645-y)
Supplement: Additional file 7: Table S6. — Abundance (percentage) of different RNAs from L.aurea CK and MJ100 degradomes. (DOC 31 kb) [file 12864_2016_2645_MOESM7_ESM.doc]

**Table S6.** Abundance (percentage) of different RNAs from CK and MJ100 *L. aurea* degradomes

| **Category** | **Unique reads (Percentage)** | | **Total reads (Percentage)** | |
| --- | --- | --- | --- | --- |
|  | **CK** | **MJ100** | **CK** | **MJ100** |
| Clean reads | 4,611,090 (100.00%) | 6,626,101 (100.00%) | 18,711,055 (100.00%) | 15,934,973 (100.00%) |
| rRNA | 22,031 (0.48%) | 25,155 (0.38%) | 673,595 (3.60%) | 495,299 (3.11%) |
| tRNA | 1,695 (0.04%) | 2,208 (0.03%) | 9,104 (0.05%) | 8,424 (0.05%) |
| snRNA | 2,173 (0.05%) | 2,936 (0.04%) | 7,333 (0.04%) | 5,651 (0.04%) |
| snoRNA | 1,874 (0.04%) | 2,512 (0.04%) | 8,245 (0.04%) | 6,602 (0.04%) |
| polyN | 1,492 (0.03%) | 2,028(0.03%) | 4,217 (0.02%) | 5,147(0.03%) |
| Mapped to reference | 2,848,018(61.76%) | 4,190,778(63.25%) | 13,311,154(71.14%) | 11,524,562(72.32%) |
| Other sRNAs | 1,733,807 (37.60%) | 2,400,484 (36.23%) | 4,697,407 (25.10%) | 3,889,288 (24.41%) |
