# Supplementary material for: Identification and differential regulation of microRNAs in response to methyl jasmonate treatment in Lycoris aurea by deep sequencing
Source: BMC Genomics. 2016 Oct 10;17:789. doi: 10.1186/s12864-016-2645-y (PMC5057397; doi:10.1186/s12864-016-2645-y)
Supplement: Additional file 9: Table S8. — List of primer sequences used for qRT-PCR experiments. (DOCX 25 kb) [file 12864_2016_2645_MOESM9_ESM.docx]

**Table S8.** List of primer sequences used for qRT-PCR experiments.

| **miRNA** | **SeqTence (5'-3')** |
| --- | --- |
| miR156a-5p | TGACAGAAGAGAGTGAGCAC |
| miR157a | TTGACAGAAGATAGAGAGCAC |
| miR159a.1 | TTTGGATTGAAGGGAGCTCTA |
| miR164a -5p | TGGAGAAGCAGGGCACGTGCA |
| miR165a -3p | TCGGACCAGGCTTCATCCCCC |
| miR166b.1 | TCGGACCAGGCTTCATTCCTA |
| miR167a-5p | TGAAGCTGCCAGCATGATCTA |
| miR172b-3p | agaaTcTTgaTgaTgcTgcaT |
| U6 snRNA | CGATACAGAGAAGATTAGCATGG |
| Uni-miR qPCR Primer | From TakaRa |
